# Bias intervention messaging in student evaluations of teaching: The role of gendered perceptions of bias

**DOI:** 10.1016/j.heliyon.2024.e37140

**Published:** 2024-08-29

**Authors:** Fiona Kim, Lisa A. Williams, Emma L. Johnston, Yanan Fan

**Affiliations:** a School of Mathematics and Statistics, UNSW Sydney, 2052, Australia; b School of Psychology, UNSW Sydney, 2052, Australia; c School of Life and Environmental Sciences, University of Sydney, 2006, Australia; d Data61, CSIRO, NSW, 2015, Australia

## Abstract

Many studies have documented discrepancies in student evaluation of teaching ratings between male and female instructors and between ethnic majority and minority instructors. Given the importance of such ratings to academic careers and the likelihood of potential intergroup bias, it is crucial that institutions consider approaches to mitigate such biases. Several recent studies have found that simple bias mitigation messaging can be effective in reducing gender and other biases. In the present research, students enrolled in several large Faculty of Science undergraduate courses at an Australian university were recruited on a volunteer basis via the course learning management system. Half of the participants were randomly assigned an intervention message highlighting potential biases relating to gender and language background. Data from 185 respondents were analysed using Bayesian ordinal regression models assessing the impact of message exposure on evaluation scores. Reading a bias intervention message caused students to significantly adjust their scores, with the nature of that change dependent on student and instructor characteristics. Among male students, the bias intervention message significantly increased scores for all except male instructors with English speaking backgrounds, for whom there was no significant impact of the message. In contrast, among female students, the bias intervention message significantly decreased scores for male instructors with English speaking backgrounds only. The sample showed an overall decrease in scores in the intervention group relative to the control group. This is the first study to detect a negative impact of bias intervention messaging on SET scores. Our results suggest students may not acknowledge their own potential bias towards instructors with whom they share similar demographic backgrounds. In conclusion, bias intervention messaging may be a simple method of mitigating bias, but it may lead to consequences in which one or more groups receive lower ratings as a result of the correction.

## 1 Introduction

In the higher education domain, student evaluations of teaching (SETs) are often used in the assessment process for promotion and performance evaluation of academic instructors. Unfortunately, a large number of studies in different settings identified systematic bias in such evaluations [1,2]. Female instructors tend to receive lower ratings than their male counterparts [[3], [4], [5], [6]]. Biases have also been documented against instructors from minority ethnic backgrounds [[7], [8], [9]]. Biases, namely those relating to gender, appear to be exacerbated in the pivot to online teaching brought about by the Covid-19 pandemic [10].

There is evidence that both female and male students exhibit implicit bias against certain groups of instructors [6]. Even in cases where gender bias is not directly observed, evidence shows that students have different expectations of male and female instructors that match culturally-conditioned stereotypes [11], such as females being seen as more interpersonally warm [12]. An experimental study found that students prefer to attend classes with instructors who possess feminine qualities (such as approachability), but expect instructors who possess masculine characteristics to be more competent [13]. Findings such as these have led a growing number of researchers to suggest that SET surveys are a flawed tool with which to assess teaching quality [2,14,15].

Given the continued use of SET data for promotion and performance evaluation, it is imperative to explore routes to mitigate systematic bias. Bias mitigation campaigns that raise awareness are a well-documented strategy for organisations looking to promote gender equity [16]. However, these strategies are not always successful, sometimes resulting in a ‘rebound effect’, in which the bias is amplified [17]. For SET surveys, simple bias intervention messaging, if it works, would be an extremely efficient and cost-effective way to mitigate potential bias. Recently, several groups of researchers have documented important new findings in bias mitigation strategies.

## 2 Literature review

### 2.1 Impact of bias mitigation messaging on SET scores

In the first study of its kind, Peterson et al. [18] conducted a field experiment in which students either completed a standard SET survey or completed the survey after being presented with a bias mitigation message. The message described the potential for gender and racial bias and the high stakes nature of SETs. The experiment was carried out in four large introductory level university courses, two in biology and two in American politics. The instructors were two male and two female instructors, all White. The message significantly improved students' ratings of female instructors but had no impact on students’ ratings of male instructors, The net effect of the message was higher ratings across the cohort. The study found no evidence to support the hypothesis that the effects of intervention are different for male and female students.

Boring and Philippe [19] compared the impact of including one of two bias mitigation messages relative to a standard SET survey. One message broadly warned students against discrimination. The other paired the warning with detailed information on documented biases in SET scores in the past at the university. This experiment was conducted at a selective French university specialising in social sciences, and the student cohort comprised of first year undergraduates. The study included a total of 155 instructors, 39% of whom were female. The study found that presenting a warning alone was not effective in reducing gender bias. However, when paired with localised evidence of prior bias, the warning message resulted in a similar pattern as the Peterson et al. [21] study. Relative to no messaging, the combination message reduced gender bias by increasing the ratings of female instructors and did not impact ratings of male instructors. In this study, however, student gender impacted the pattern of results. The effect of the combined message was driven by male students evaluating female instructors more favorably; the message did not significantly impact ratings by female students.

To date, only one study has accounted for ethnicity and gender of both instructors and students. Genetin et al. [20] assessed the impact of three types of bias mitigation messages, separating components of the message used by Peterson et al. [18]: a message that described the potential for gender and racial bias, a message detailing the high stakes nature of SETs, and a combination message drawing from both. The study was conducted at a US university and involved roughly 400 instructors. Importantly, their analyses explored the impact of the evaluation as a function of student and instructor gender as well as student and instructor ethnic background. Neither message describing potential bias significantly impacted instructor ratings. The only observed effects were a function of the high-stakes (only) message, where female racial/ethnic minority instructors were rated more highly following the high-stakes message relative to the control message. This effect was predominantly driven by higher ratings from female minority students, suggesting an affinity effect [20]. The authors concluded that there is no negative effect on instructor ratings as a result of bias mitigation messages.

These past studies highlight that the effects of bias mitigation messaging are variable. Reduced gender bias depends on message wording and, in some cases, instructor and student characteristics. Of note, reduced gender bias observed in these studies has taken the form of increased ratings for female and ethnic minority instructors, without impacting the ratings for male instructors.

### 2.2 Theoretical background

This research is grounded in classic theories regarding intergroup bias. Intergroup bias captures the systematic tendency to evaluate groups to which one belongs (in-groups) more favorably than groups to which one does not belong (out-groups) [[21], [22], [23]]. Such evaluative biases exist for both groups as a whole as well as members of those groups, and can take the form of favoring the in-group and/or derogating the out-group. Several theories outline the specific nature, origins, and mechanisms of intergroup bias, including Social Identity Theory [24], Optimal Distinctiveness Theory [25], and Social Dominance Theory [26]. These theories share a common theme that underscores the predominance of group-based social perception, and how such perceptions guide outcomes according to group membership.

Any group membership can give rise to intergroup bias. However, gender is a highly salient group cue via which people categorise one another into groups and around which intergroup bias arises. Gender categorisation arises early in development [27] and permeates social cognition among adults [28,29]. Sexism, defined as attitudes, beliefs, or behaviors that support inequality between men and women [30], is fed by gender-based intergroup perception and bias. Of relevance to the present research, sexism and gender bias are underpinned by durable stereotyped beliefs about the traits and abilities of men and women [31,32]. Generally, women are stereotyped to be warm, nurturing, and friendly. Men, in contrast, are stereotyped to be competent, intelligent, and of high status. Such stereotypes form the basis of behavioural expectations: women and men are expected to behave in stereotype consistent ways. When they do not, they are derogated and face backlash [33,34]. In higher education settings, the status and competence differential between students and instructors is likely to elicit a context in which female instructors are perceived to be counter-stereotypical and thus are more harshly judged. Scientific fields are likely host to exacerbated effects along these lines, given the gender stereotyping regarding science as a ‘male career’ [35,36].

Ethnicity, cultural background, and – as a cue to these – language background are also highly salient group differentiating characteristics. As is the case with gender, race and ethnicity are salient cues to group membership among both children [37] and adults [28,29]. The content of stereotypes relating to race and ethnicity vary by particular group and culture [38], but evidence suggests a global pattern in which minority racial and ethnic groups are perceived to be lower in competence and/or warmth than majority groups, who are stereotyped to be high on both dimensions [39]. Following a similar logic as detailed above regarding gender, ethnic and racial minority instructors may face backlash, and thus be more harshly judged [40]. In Western countries, this is likely to be especially true in science fields, in which researchers and instructors are predominantly White and have English as their primary language [[41], [42], [43]].

Group-based biases are prevalent but are by no means inevitable. One popular approach to mitigating group-based bias is to raise peoples’ awareness of its potential to evoke motivated self-regulation. Per the Self-Regulation of Prejudiced Responses Model [44], highlighting discrepancies between how a person believes they ought to act and the stereotyped and prejudicial ways in which they (might) act motivates bias reduction. This approach works best among people who believe themselves to be and/or value being unbiased [45]. In the SET context, bias mitigation messaging approaches leverage such ideas to seek to promote more equitable outcomes according to group memberships such as gender and ethnicity and cultural background [[18], [19], [20]].

The present research aimed to evaluate the impact and effectiveness of bias mitigation messaging in the Australian university SET context. Given prior research documenting gender and instructor language background biases at the university [7,12], our bias mitigation message emphasized the bias that might arise from gender and/or instructor language backgrounds (replacing racial bias as referenced in prior bias mitigation studies). We explored the impact of the message according to instructor gender and language background, and student gender. Mirroring the design of Peterson et al. [18], students either completed a teaching evaluation survey after reading a message about bias and the high stakes nature of SETs, or completed the survey in a standard (no-message) context. This experiment was carried out across multiple undergraduate courses in a Faculty of Science within a large public university in Australia. The sample involved both male and female instructors, male and female students, and instructors from a range of language backgrounds.

## 3 Methods

This research was approved on the June 18, 2020 by the UNSW Human Research Ethics Committee (Approval #HC200203).

### 3.1 Data collection

Given that SET survey outputs are still used widely in high stake performance evaluations within the university, any experiments involving these surveys need to avoid unintended adverse outcomes for instructors. Consequently, this experiment was carried out independently of the formal course and teaching evaluations run by the university, which typically occur at the conclusion of a course. Our survey was designed to mimic the official SET surveys but was administered in the middle of a ten week teaching term.

The experiment was carried out in large courses (enrolments exceeding 100 students) within the Faculty of Science. Previous analysis of historical SET data from this Faculty documented discrepancies in ratings attributed to gender or cultural biases [7].

Instructors in courses meeting the criteria (i.e., large enrolment courses in the Faculty of Science) were invited to opt in to their course being included in the experiment on a voluntary basis. Instructors who agreed to take part in the experiment were asked to complete a short survey (see Appendix A), which included questions regarding gender and language background of instructors (English as a primary language vs. English as a secondary language). The 18 courses involved in the experiment were from the following Schools: Biotechnology and Biomolecular Sciences; Biological, Earth and Environmental Sciences; Mathematics and Statistics; Physics; and Psychology. These courses included a total of 21 instructors (due to the team-teaching model at the university). Of the 21 instructors represented in the data, 9 were female and 4 reported English as a secondary language (2 of these were female).

Student participants were recruited through an announcement posted on each participating course's online learning management system. Participation in the experiment was presented as strictly voluntary, and students were free to withdraw at any time. The student survey contained questions that appear in the official SET surveys at this university (see Appendix B). Students rated their course and instructor/s on a variety of metrics. We primarily focused on responses to the question: “Overall, I am satisfied with the quality of this person's teaching” (henceforth referred to as overall instructor satisfaction). We also analysed responses to the question: “Overall, I am satisfied with the quality of the course” (henceforth referred to as overall course satisfaction). Responses to these two questions were made on a Likert-style scale ranging from 1 (“strongly disagree”) to 6 (“strongly agree”). Students had the option to provide written comments to open-ended questions on the best features of the instructors' teaching and suggestions for improvements. Respondents also provided demographic information (e.g., gender).

Randomisation was carried out at the student level, where each student was designated to receive the bias mitigation intervention message, or not, with equal probability. Half of the participating students were designated to receive the bias mitigation message immediately before completing the survey. The bias mitigation message was adapted from Peterson et al. [18] to better suit the Australian context by citing language background as an additional source of implicit bias. The message read:

“*Student evaluations of teaching play an important role in the review of teaching staff.*

Your opinions are incorporated into the periodic review of teaching staff. [Institution Name] recognises that student evaluations of teaching are often influenced by students' unconscious and unintentional biases about the background and gender of the teaching staff. Various studies have shown that women and teaching staff of non-English speaking background are systematically rated lower in their teaching evaluations than men of English speaking background, even when there are no actual differences in the teaching or in what students have learned.

As you fill out this survey please keep this in mind and make an effort to resist stereotypes about university teaching staff. Focus on your opinions about the content of the course (the assignments, the textbook, the in-class material) and not unrelated matters (the instructor’s appearance).”

Overall, 185 students participated in the experiment, with some students rating multiple instructors in the same course due to the team-teaching model at the university. In these cases, the student was assigned the same message condition for all surveys. In total, this design resulted in 281 individual rating surveys. A breakdown of the number of responses according to message condition and instructor and student characteristics is presented in Table 1. The size of the dataset was constrained by the number of courses with instructor consent and the number of students volunteering to participate. We acknowledge the relatively small sample size, and the fact that inference of observed small effects might be statistically inconclusive. We provide a detailed description of our inference approach in the next section.

**Table 1:** The number of survey responses broken down by demographic characteristics of the students and instructors who participated in the experiment.

	Control Condition	Intervention Condition
**Instructor Gender**		
Female	58	59
Male	82	82
**Instructor Language Background**		
English as a Primary language	112	119
English as a Secondary language	28	22
**Instructor Ethnicity**		
White	123	126
Non-White	17	15
**Student Gender**		
Female	111	90
Male	29	51

### 3.2 Statistical analysis

We carried out statistical analysis using an ordinal regression framework [46]. In particular, we used the cumulative logit link model of the form

(1) $$\log\;\left(\frac{P(\left(y_{stc}\leq j\right)}{1-P\left(y_{stc}\leq j\right)}\right)=\theta_{j}-X_{i}^{T}\beta \;-\;\alpha_{t,c}$$

where *j* = 1*, …,* 6 refers to the response levels, *P* ($y_{stc}$ ≤j) is the probability of student *s* from course *c* taught by instructor *t* giving a score less than or equal to *j*, given $X_{i}$ = ($x_{1i}$*, …,* $x_{pi}$) the vector of fixed effect measurements (such as student and instructor characteristics) and $\alpha_{t,c}$ is the vector of random effects coefficients, modelling the dependence when a survey is completed for the same instructor in the same course. These random effects also served to account for any variation that was not captured by the fixed effects parameters β = ($\beta_{1}$*, …,* $\beta_{p}$). We did not model the additional effects of multiple student surveys, as these only involved a small number of participants and most of these responded to only two surveys. That is, due to the small sample size, we did not model student-specific random effects. Finally, the parameters $\theta_{j}$ are constant terms corresponding to each response level *j*. We adopted a Bayesian statistical approach, where the use of Markov chain Monte Carlo (MCMC) methods enabled precise calculations of confidence intervals. For small sample sizes (such as in this study), this approach is more accurate than alternative approaches that rely on large sample asymptotic results.

The *brms* [47] package was used in R [48] to specify a Bayesian ordinal regression model. *brms* utilises *Stan*, which is a C++ package used to perform full Bayesian inference [49]. *Stan* implements Hamiltonian Monte Carlo (HMC) and its extension, the No-U-Turn Sampler (NUTS) [49], where inference is based on the posterior distribution arising from the combination of the likelihood and the prior. Under the Bayesian paradigm, we assigned a prior to the parameters *θ*_*j*_, *β*, *α*_*t,c*_. The half Student-t prior with 3 degrees of freedom was used, since this leads to better convergence than a half Cauchy prior, whilst also being relatively weakly informative [47]. Sensitivity analyses showed that this prior is fairly robust and produces point estimates which are similar to those obtained through frequentist statistical approaches.

Here, we report the posterior means of the model parameters from MCMC, as well as the upper and lower 0.05 quantiles of the posterior distribution as the 90% credible interval for these parameters. This level was selected to enable detection of significant effects that may be present at a higher credible interval with a larger sample size. Significance of a covariate was determined when the value of zero was not contained within the lower and upper bounds.

The response variable $y_{stc}$ comprised the overall satisfaction of the instructor's quality of teaching as assessed by the students. After initial exploratory analyses to refine the regression model, the explanatory variables were: a dummy variable which indicated whether the student was presented with a bias mitigation intervention message (or not) prior to completing the survey (INT); student gender (1 if male, 0 otherwise), instructor gender (1 if female, 0 otherwise), and instructor language background (1 if primary language was not English, 0 otherwise). We also included the two-way interaction terms between these four variables. Higher order interactions involving the intervention term were not significant, so were excluded from the final model.

The model was run with 4 separate MCMC chains of different starting values. Each chain was run for 10,000 iterations discarding the initial warm-up period of 2000 iterations. To check for MCMC convergence, the $\hat{R}$ (potential scale reduction factor) were examined with $\hat{R}$ around 1.00 implying convergence [50]. We carried out convergence assessments based on $\hat{R}$ values as well as visual examination of the traceplots.

We interpret the estimates of the fixed effect coefficients β. While random effects $\alpha_{t,c}$ can be used to compare individual instructor effects, this was not the goal of this analysis, and we used them only to account for the dependencies between survey responses. $\theta_{j}$ s are intercept terms for each response level. These were not of interest for establishing the results of the experiment. For a detailed interpretation of the coefficients β, the reader can refer to Ref. [7]. Strictly speaking, they indicate the log-odds of getting higher scores (response y) relative to a baseline group. However, the reader can interpret these coefficients as the relative contributions to the scores associated with belonging to a given group.

## 4 Results and discussion

### 4.1 Regression modelling results for instructor satisfaction ratings

**Fig. 1:**
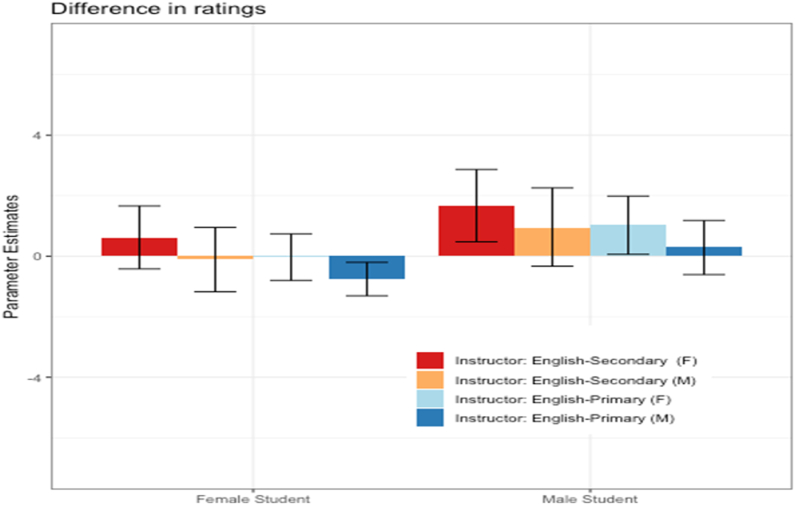
The effects of the bias mitigation message intervention relative to control, by instructor and student demographics. Results are presented separately for combinations of instructor gender (male and female) and language background (English as a primary language background, English as a secondary language background), and student gender (male and female). The bars indicate differences of coefficient effects between the intervention condition and the control condition, and the whiskers indicate 90% confidence intervals. A positive value indicates relatively higher ratings in the intervention condition relative to the control condition. Significant effects of condition can be inferred if the whiskers for a particular group do not cross zero.

**Fig. 2:**
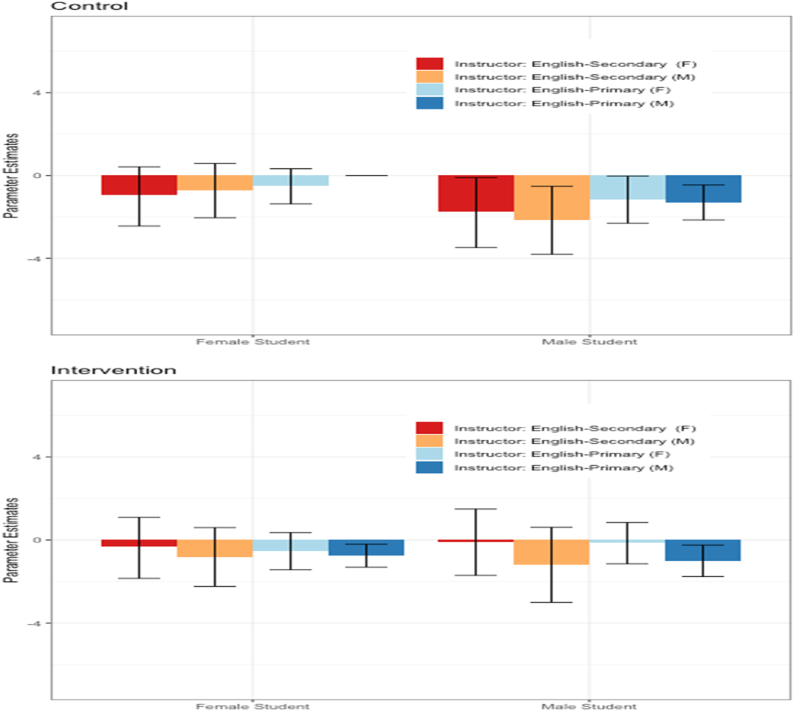
Posterior mean of the coefficient parameter for each instructor and student group in the control (top) and intervention (bottom) conditions, separately for each of the student and instructor demographic combination. Whiskers indicate 90% confidence intervals. Significant differences from the reference group (female students rating male instructors with English as a primary language in the control condition) can be inferred if the whiskers for a particular group do not cross zero.

Results of the regression model for instructor satisfaction ratings are provided in Table 2. The coefficients for the intervention condition, student gender, and the interaction between the intervention condition and student gender were all statistically significant at the 90% level. However, these outputs are hard to interpret, as the parameter estimates are relative to the reference group of female students evaluating male instructors with English as their primary language background. Fig. 1, Fig. 2 facilitate interpretation of these results.

Fig. 1 shows the net effect of the bias mitigation intervention message for each combination of student gender, instructor gender, and instructor language background. This was obtained by calculating the difference in the covariate effects β between the control condition and the intervention condition, separately for student and instructor characteristics. The 90% confidence intervals (vertical lines) were obtained by summing the relevant β coefficients in the intervention condition and subtracting the corresponding coefficients in the control condition based on the MCMC outputs. The confidence interval bars reflect the estimation uncertainty and thus signify the results as significant for that group if the whiskers do not cross zero.

The bias mitigation intervention message resulted in significantly higher ratings by male students (Appendix C, Table 2). From Fig. 1, we see that this effect varied according to instructor language background and instructor gender. Ratings by male students were significantly higher in the intervention condition relative to the control condition for female instructors, with a larger effect for female instructors with English as a secondary language. Ratings by male students for male instructors with English as a primary language and male instructors with English as a secondary language did not significantly differ according to intervention condition.

A different pattern emerged among female students. Ratings by female students were significantly lower in the intervention condition relative to the control condition for male instructors with English as a primary language. The intervention did not significantly impact ratings by female students of other instructor groups.

To gain further insight into the impact of the intervention, we plotted the coefficient effects in the control and intervention conditions separately in Fig. 2. We use female students evaluating male instructors with English as a primary language in the control condition as the reference group for the following. First focusing on the control condition, female students’ ratings of female instructors and instructors with English as a secondary language did not differ from their ratings of male instructors with English as a primary language (i.e., the reference group). Male students, however, gave significantly lower ratings to all instructor groups, including male instructors with English as a primary language, relative to the reference group. Turning to the intervention condition, male students rated male instructors with English as a primary language significantly lower than the reference group. Female students in the intervention condition rated male instructors with English as a primary language significantly lower than the reference group. Differences for all other student and instructor demographic combinations between the intervention condition and the reference group were not statistically significant.

One possible explanation for the differences in the impact of the intervention between male and female students could be that, upon encountering the bias mitigation message, male students seek to reduce biases by elevating otherwise lowered ratings of female instructors and male instructors with English as a secondary language (the groups purportedly negatively impacted by bias). Female students, in contrast, may seek to reduce biases by reducing ratings of male instructors with English as a primary language (the group purportedly positively impacted by bias). Further experiments are needed to explore underlying beliefs and possible motivations of the observed differing directional change. Theoretical models such as the Flexible Correction Model [51], which incorporates people's lay theories about the origins of bias in predicting outcomes of efforts to reduce bias, will be useful in guiding such work.

To our knowledge, this is the first documented decrease in ratings as a function of bias mitigation messaging in the context of SETs. Significant changes in instructor ratings as a function of bias mitigation messaging in past research have been carried by increased ratings of instructors historically negatively impacted by bias (e.g., female instructors, instructors of ethnic/racial minorities [[18], [19], [20]]). Note that reduced ratings for male instructors has been documented as an outcome of a different bias mitigation intervention: a self-affirmation task [52], in which the authors showed that contemplating aspects of one's self that positively contribute to one's self-image eradicated otherwise observed gender biases in SET scores. While such outcomes may not be palatable (especially to male instructors with English as a primary language), the net effect is reduced gender and language background disparities. Because there is no ‘true score’ in this context [52], the aim of bias mitigation is reduction in differences according to demographic features.

In another departure from prior research, we did not see evidence of an affinity effect on the basis of gender as documented in prior work [20]. In the present research, the only significant effects of the intervention were observed in cross-gender pairings (lower ratings of male instructors with English as a primary language by female students and higher ratings of female instructors with English as a secondary language by male students).

**Fig. 3:**
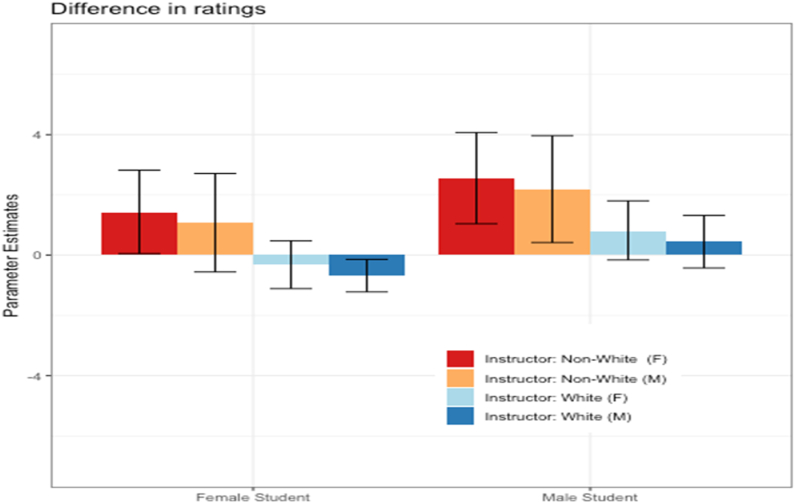
The effects of the bias mitigation message intervention relative to control, by instructor and student demographics. Results are presented separately for combinations of instructor gender (male and female) and ethnicity (White and non-White), and student gender (male and female). The bars indicate differences of coefficient effects between the intervention condition and the control condition, and the whiskers indicate 90% confidence intervals. A positive value indicates relatively higher ratings in the intervention condition relative to the control condition. Significant effects of condition can be inferred if the whiskers for a particular group do not cross zero.

To align with prior research on the effects of bias mitigation messaging as a function of instructor ethnicity, we implemented a model with instructor ethnicity (White vs. non-White) replacing instructor language background. While the intervention didn't mention ethnicity, it is possible that biases stemming from this demographic characteristic might also be reduced as a function of the intervention. Table 1 presents the breakdown of the number of survey responses by instructor ethnicity. Results of this analysis should be interpreted with caution due to the relatively smaller number of non-White instructors. Appendix C, Table 3 presents the results from the ordinal regression model examining the influence of student gender, instructor gender, and instructor ethnicity on instructor satisfaction ratings as a function of condition. Significant interactions between condition and student gender and between condition and instructor ethnicity were observed. The effects of the intervention by demographic group are presented in Fig. 3. Mirroring the effects observed with instructor language background, female students rated male White instructors lower and male students rated female non-White instructors higher in the intervention condition than the control condition. Unique effects also emerged: female students rated female non-White instructors higher and male students rated male non-White instructors higher in the intervention condition than the control condition.

Overall, we found that the impact of a bias mitigation message on instructor satisfaction ratings depended on student gender, instructor gender, and instructor language (or ethnic) background. When rating male instructors with English as a primary language or of White ethnicity, female students significantly reduced their rating following exposure to the bias mitigation message. To our knowledge, this is the first intervention message experiment to note this effect and extends past literature where no negative impacts were found for male instructors [18,20]. Male students, in contrast, responded to the intervention by increasing their ratings of female instructors or non-White ethnicity instructors. Thus, while the intervention message did bring about a reduction in net differences in instructor ratings based on gender and other demographic characteristics, the negative impacts found for male instructors caution the use of bias mitigation messaging. The differing pattern of responses observed for male and female students suggests that there are differences in their perception of how bias manifests and how it might be rectified. It is clearly less than straightforward to mitigate biases that emerge in SET data. University administration should consider the weight, if any, they put on academic performance as indexed by SET survey data.

### 4.2 Results based on open-ended comments

Due to relatively few students opting to leave comments to the open-ended questions regarding the best features of instructors’ teaching and suggestions for improvements, comprehensive analysis of these data was precluded. Here, however, we provide some overarching observations on the nature of the comments as a function of student gender and intervention condition.

The bias mitigation intervention impacted whether or not male students provided comments. In the control condition, 38% of male students provided comments. However, in the intervention condition, 72% of male students left comments. Response rates did not differ for female students across conditions (control: 49%; intervention: 48%).

Most responses were in relation to the question regarding best features rather than improvements. Using a high-level assessment of content, female and male students commented on different aspects of instructors' teaching. Female students' comments tended to focus on instructors' engagement, kindness, knowledge, and passion. Male students’ comments were more varied in their content, and cited more tangible qualities of teaching such as organisation and effort. There were no discernible differences in the content responses as a function of the intervention.

We point to other comprehensive analysis of open-ended comments in SETs, many of which document systematic biases against women and people of culturally diverse backgrounds [12,19,53]. More research is needed to assess the impact of bias mitigation interventions on open-ended comments. Such research should utilise large datasets where possible and might effectively make use of text mining, topic analysis, and sentiment analysis methodologies [54].

### 4.3 Limitations

While this study offers several strengths including sampling from a range of science disciplines and providing a test of a bias mitigation message in an Australian context (prior interventions were deployed in the United States and Europe [[18], [19], [20]]), we acknowledge several limitations. First, the sample size for both instructors and students was modest. Our sample included particularly few instructors with English as a secondary language. Findings for this demographic group may not be very representative of the broader population. We were also unable to explore the effect of the students’ language background on the intervention due to lack of data. Further study with a larger sample size and comprehensive demographic data would enable more confident assessment of the impact of bias mitigation interventions. This is particularly relevant for universities with demographically diverse instructors and to answer broader calls to move beyond binary considerations of gender in intergroup bias research [55]. Further research is also needed to establish the efficacy of interventions in non-science disciplines and in non-lecture teaching formats (e.g., seminars and other small-group teaching). Finally, further experiments may enable richer analysis of the impact of bias mitigation messages on the rate and content of student comments.

**Fig. 4:**
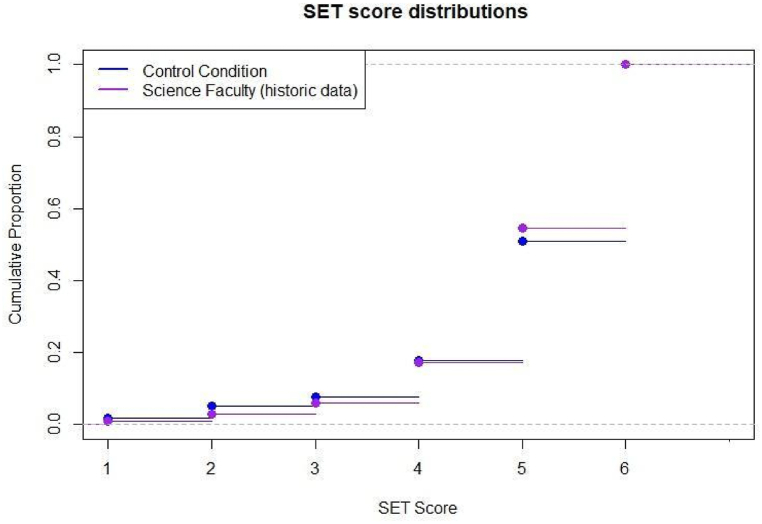
Empirical cumulative distribution plots of the responses provided in the control condition of the present research compared with historical responses from the Faculty of Science in which the research was conducted.

One concern with small sample sizes is self-selection and non-representation. The response rate of this study (4%) is indeed lower than the historic response rates in the Faculty of Science at the university in which the experiment was conducted, which ranged between 25 and 30%. To address concerns of self-selection and non-representation stemming from the small sample size, we performed a series of two-sample Kolmogorov-Smirnov tests, which tests for statistical differences between the response distributions of any two samples. We compared the response scores of the control condition acquired from our survey (*N* = 118) and historical responses within the Faculty of Science (based on Semester 2 of 2016, the most recent historical data to which we have access, *N* = 3453). The empirical cumulative distribution functions of each group are plotted in Fig. 4. The results showed p-value *p* ≈ 1, thus providing no evidence to suggest that the distribution of the sample in our control condition is different to the historical data in the Faculty of Science. Similar results hold when comparing data from the control condition to the whole university (*p* = 0.59; *N* = 16*,* 903). Likewise, distributions for male instructors (*N* = 82 for our control condition and *N* = 2*,* 179 from historical Faculty of Science data) and female instructors (*N* = 58 for our control condition and *N* = 1*,* 274 from historical Faculty of Science data) were similar, *p* ≈ 1 and *p* = 0.75, respectively. While these results assuage concerns regarding self-selection and associated biases in observed results, caution is needed in generalising conclusions until replication studies with larger samples are carried out.

Another limitation of this research relates to experimenter demand effects, where participants may change their behaviour due to cues about what constitutes appropriate behaviour in the context of research [56]. In this experiment, students were informed that their responses would assist researchers to better understand the student experience, which mirrors the messaging given to students in standard SET survey processes. As such, our study mimics the real-life context of SET surveys and overall demand effects in this view would have been minimal. With regard to the intervention condition specifically, expected intervention effects could in fact be framed as demand characteristics: students exposed to the intervention message, which alerted them to the potential for bias, would plausibly deduce that they were expected to adjust their ratings so as to reduce bias. Both success of the intervention and demand characteristics would result in the same outcome: reduced bias. That said, the heterogeneous nature of intervention effects according to student and instructor characteristics we observed are difficult to reconcile with an interpretation solely through the lens of demand characteristics.

### 4.4 Implications

Although bias mitigation message interventions are relatively simple to implement, the practical implications of this work is clear. The impact of such interventions for different instructor groups and among different student cohorts needs to be better understood before widespread rollout.

In the meantime, this research underscores the need to consider the weight (if any) placed on SET data in evaluations of university instructor teaching quality. Researchers have called for relegating the use of SET data for formative, rather than summative, purposes [57]. Evaluations of teaching effectiveness might instead rely on a multicomponent approach including peer evaluations of teaching and portfolios [58], and assessments of pre- and post-course learning [59].

This research underscores the nuanced nature of intergroup processes as has long been theorised by social psychologists [[21], [22], [23]]. Intergroup biases stem from a variety of group categorisations, and sometimes simultaneously so. Moreover, relative group memberships between perceivers and their targets (in the case of SETS: students and their instructors) is essential. This research also underscores that theories of motivated self-regulation for prejudice reduction [44] can be translated to practice, though also presents a call for new theoretical approaches that can fully capture the complexity of intergroup dynamics at play in student-instructor judgments.

## 5 Conclusion

This study exposed students to a bias mitigation intervention message warning them against the biases commonly found in the SET surveys they were about to complete. The goal of the study was to provide insight into the effect of the intervention on the students’ ratings of their instructors in the Australian university context, incorporating both instructor and student demographics. Results suggested that the bias mitigation intervention message caused female students to give lower ratings to male instructors with English as a primary language and caused male students to give higher ratings to female instructors.

While the intervention was effective in persuading students to adjust their ratings in some cases, it also led to lower overall instructor satisfaction ratings across the cohort, and lower scores for male instructors with English as a primary language background. Policy makers should thus take caution when considering the use of bias mitigation messaging. Although simple to implement, the implications for different instructor demographics need to be better understood before widespread rollout. In the meantime, this research underscores the need to consider the weight placed on SET data in evaluations of university instructor teaching quality.

## Funding

The authors did not receive financial support from any organization to produce the specifics of the submitted work.

## Ethics approval

This research was approved by the UNSW Human Research Ethics Advisory Panel (HREAP), HC200203.

## Consent to participate

All participants provided informed consent to participate in this study in accordance with the ethics guidelines and approvals.

## Consent for publication

Publication of the work is supported by the authors and in accordance with the ethics guidelines set.

## Data availability statement

Data and code are available publicly at

https://github.com/yananfand61/Gender-and-Cultural-bias-in-SET.

## CRediT authorship contribution statement

**Fiona Kim:** Writing – original draft, Visualization, Methodology, Investigation, Formal analysis, Data curation, Conceptualization. **Lisa A. Williams:** Writing – review & editing, Validation, Data curation, Conceptualization. **Emma L. Johnston:** Writing – review & editing, Supervision, Conceptualization. **Yanan Fan:** Writing – review & editing, Visualization, Investigation, Formal analysis, Conceptualization.

## Declaration of Competing interest

The authors declare that they have no known competing financial interests or personal relationships that could have appeared to influence the work reported in this paper.

## Figures and Tables

**Table 2:** Posterior mean and 90% credible interval from the ordinal regression model with instructor language background.

Variable	Estimate	CI Lower	CI Upper
**INT**	**−0.75**	**−1.31**	**−0.20**
**Male Student**	**−1.30**	**−2.15**	**−0.46**
Female Instructor	−0.49	−1.37	0.33
Instructor ENG Secondary	−0.70	−2.04	0.59
**INT x Male Student**	**1.06**	**0.11**	**2.00**
INT x Female Instructor	0.72	−0.13	1.57
INT x Instructor ENG Secondary	0.64	−0.40	1.69
Male Student x Female Instructor	0.64	−0.31	1.61
Male Student x Instructor ENG Secondary	−0.15	−1.39	1.09
Female Instructor x Instructor ENG Secondary	0.26	−1.58	1.92

**Table 3:** Posterior mean and 90% credible interval from the ordinal model with instructor ethnicity.

Variable	Estimate	CI Lower	CI Upper
INT	1.06	−0.56	2.71
Male Student	−1.37	−3.32	0.60
Female Instructor	−0.26	−1.12	0.56
Instructor White	0.96	−0.45	2.50
**INT x Male Student**	**1.13**	**0.20**	**2.06**
INT x Female Instructor	0.36	−0.53	1.26
**INT x Instructor White**	**−1.74**	**−3.28**	**−0.20**
Male Student x Female Instructor	0.63	−0.41	1.67
Male Student x Instructor White	−0.01	−1.81	1.72

## Appendix 1

### Appendix A 1.1 Instructor Survey Questionnaire

The instructors completed the following survey.

1. Please enter your name
2. Please select all your courses from the list below
3. Please indicate the gender which you identify
  • Male
  • Female
  • Non-binary/Gender non-conforming
  • Prefer to self-describe [open box]
  • Prefer not to say
4. With which ethnic group(s) do you identify? (select all that apply)
  • African
  • Asian (including East Asia, Southeast Asia, and South Asia)
  • Black or African-American
  • Hispanic or Latino/a/x
  • Indigenous Australian or Torres Strait Islander
  • Native American
  • Persian or Arabic
  • Pacific Islander
  • White or Caucasian or European
  • Another ethnic group not specified (enter if you wish):
  • Prefer not to say
5. Were you born in Australia?
6. Were your parents born in Australia? [One/Both/Neither]
7. Is English your primary or secondary language? [Primary/Secondary]
8. How many times have you previously taught this course?
9. How many years have you been engaged in university-level teaching?

### Appendix B 1.2 Student Survey Questionnaire

The students completed the following survey: Please provide a score between 1 and 6 for the course and teacher questions, where 1 = Strongly Disagree 2 = Disagree 3 = Moderately Disagree 4 = Moderately Agree 5 = Agree 6 = Strongly Agree.

#### Course Questions

1. I feel part of a learning community.
2. The feedback provided helps me learn.
3. The course resources help me learn.
4. The assessment tasks are relevant to the course content.
5. Overall, I am satisfied with the quality of the course.
6. What are the best things about this course? [Open comments]
7. What can be improved? [Open comments]

#### Instructor Questions

1. Please select one of the instructors for this course to evaluate [Students will be provided with a list of all instructors in the course and an option to select if they do not know the name of the instructor]
2. This teacher encourages student participation.
3. This teacher provides helpful feedback.
4. Overall, I am satisfied with the quality of this person's teaching.
5. The best features of this person's teaching are … [Open comments]
6. This person's teaching can be improved by … [Open comments]

#### Student Information

1. Please indicate the gender which you identify.
  • Male
  • Female
  • Non-binary/Gender non-conforming
  • Prefer to self-describe [open box]
  • Prefer not to say
2. Are you an international student? [Yes/No/Prefer not to say]
3. What year of university are you currently undertaking? [1/2/3/4/5+]
4. Please estimate your Weighted Average Mark (WAM) for this term?/What is your current WAM to date? (dependent on answer to Q3) [0–100]
5. Please estimate the mark you will receive in this course [0–100]
6. With which ethnic group(s) do you identify? (select all that apply)
  a. African
  b. Asian (including East Asia, Southeast Asia, and South Asia)
  c. Black or African-American
  d. Hispanic or Latino/a/x
  e. Indigenous Australian or Torres Strait Islander
  f. Native American
  g. Persian or Arabic
  h. Pacific Islander
  i. White or Caucasian or European
  j. Another ethnic group not specified (enter if you wish):
  k. Prefer not to say
